# Investigating changes in mental health services utilisation in England and their impact on health outcomes and wellbeing during the COVID-19 pandemic: Protocol for a health data-linkage study

**DOI:** 10.1371/journal.pone.0283986

**Published:** 2023-04-06

**Authors:** Ge Yu, Luke Vale, Peter McMeekin, Sheena E. Ramsay, Yu Fu

**Affiliations:** 1 Population Health Sciences Institute, Newcastle University, Newcastle upon Tyne, United Kingdom; 2 NIHR Applied Research Collaboration North East and North Cumbria, St Nicholas’ Hospital, Gosforth, Newcastle Upon Tyne, United Kingdom; 3 Faculty of Health and Life Science, Northumbria University, Newcastle upon Tyne, United Kingdom; 4 Department of Health Services Research, University of Liverpool, Liverpool, United Kingdom; Brunel University London, UNITED KINGDOM

## Abstract

**Introduction:**

Linking routinely collected health care system data records for the same individual across different services and over time has enormous potential for the NHS and its patients. The aims of this data linkage study are to quantify the changes to mental health services utilisation in responses to the COVID-19 pandemic and determine whether these changes were associated with health-related outcomes and wellbeing among people living in the most deprived communities in North East and North Cumbria, England.

**Methods and analysis:**

We will assemble a retrospective cohort of people having referred or self-referred to NHS-funded mental health services or Improving Access to Psychological Therapies (IAPT) services between 23^rd^ March 2019 and 22^nd^ March 2020 in the most deprived areas in England. We will link together data from retrospective routinely collected healthcare data including local general practitioner (GP) practice data, Hospital Episode Statistics admitted patient care outpatients, and A&E, Community Services Data Set, Mental Health Services Data Set, and Improving Access to Psychological Therapies Data Set. We will use these linked patient-level data to 1) describe the characteristics of the cohort prior to the lockdown; 2) investigate changes to mental health services utilised between multiple time periods of the COVID-19 lockdown including out of lockdown; 3) explore the relationship between these changes and health outcomes/wellbeing and factors that confound and mediate this relationship among this cohort.

**Strengths and limitations of this study:**

- This study comprises a deprived population-based cohort of people having referred or self-referred to NHS-funded secondary mental health services or Improving Access to Psychological Therapies (IAPT) services over an extended period of the lockdown in England (2019–2022).
- This study will utilise a new longitudinal data resource that will link together detailed data from a cohort of individual participants and retrospective administrative data relating to the use of primary, secondary, and community care services.
- The study period covers pre-lockdown, different lockdown and post-lockdown, and out of lockdown periods up to March 2022.
- Routinely collected administrative data contain limited contextual information and represent an underestimate of total health outcomes for these individuals.
- Routinely collected datasets can often been incomplete or contain missing data, which can make it difficult to accurately analyse the data and draw meaningful conclusions.
- Intervention and treatment for mental health conditions are not wholly captured across these data sources and may impact health outcomes.

## Introduction

The coronavirus disease 2019 (COVID-19) has increased the risk of mental health problems and it is estimated that the pandemic will lead to new or additional mental health support for up to 10 million people in England (around 20% of the population) [1]. Evidence from previous studies illustrated that the virus and the lockdown deteriorated population mental health and disproportionately worsened the mental health burden for more deprived population [2,3]. People living in the most deprived areas can face various barriers to accessing mental health services [4–6]. Mental healthcare adaptations for infection-control reasons could have been disproportionally detrimental to them after the first UK-wide lockdown began on the 23 March 2020 due to difficulties attending review appointments in person and closure of support services [7]. This can have a significant negative impact on their heath, on their partners and the wider families, and on the society as a whole [8–10]. The unequal impact of the pandemic is likely to entrench and exacerbate the existing structural inequalities in mental health among the most deprived communities, and services provided failed to meet their increasing needs for mental health conditions in the time of the COVID-19 pandemic [11].

However, there is an incomplete picture of 1) the actual use of mental health services by deprived population, and 2) the impact of the COVID-19 pandemic on their mental health service utilisation. Such information are essential to inform current policies which envisage an unprecedented expansion of specialist mental health as part of the Five Year Forward View for Mental Health in England [12] and the National Health Service (NHS) Long Term Plan [13]. The pandemic could provide an opportunity to rethink conventional approaches to mental health services planning to meet patients’ needs. For example, remote community treatment and support has long been suggested, but has not previously been implemented widely because of barriers and challenges from both healthcare staff and service users. Since the onset of the pandemic, the situation has changed [14]. Similarly, the threshold for hospital admission for mental illness varies between individuals and requires continuous adaptation over time. Therefore, learning from service utilisation changes due to the COVID-19 pandemic, and their consequences for people’s physical and mental health is vital to inform policy solutions for integrated service recovery and effectively plan services that reach those with the greatest needs.

### Aim

Population-based observational studies using routinely collected healthcare data allow analysis of variation in service use and outcome to be studied. This data linkage study aims to examine the impact of the pandemic on patterns of NHS services utilisation for people from the most deprived communities in North East and North Cumbria, England, and determine whether these patterns were associated with health-related outcomes.

### Objectives

The specific objectives of this study are:

1. To investigate NHS service utilisation (i.e., settings and pathways of care) where people were in contact with NHS-funded secondary mental health services in the year prior to the lockdown across the deprived population and in specific groups such as the elderly or ethnic minorities.
2. To quantify changes in their mental health service utilisation between multiple time periods across pre-, during- and post-lockdown including out of lockdown in England.
3. To identify the specific factors at the individual and general practitioner (GP) -levels that contribute to differences in the use of mental health services.
4. To quantify patients’ health outcomes among the cohort between the multiple time periods.
5. To estimate the associations between mental health service utilisation and patients’ health outcomes
6. To explore the contributions of individual- and GP practical-level factors to associations between mental health service utilisation and health outcomes, establishing when, where and for whom mental health services may be effective.
7. If data is available, to conduct comparative analyses using data from the less deprived communities in North East and North Cumbria, England.

## Methods and analysis

### Design

This retrospective study will link seven routinely collected healthcare data sets in England between March 2019 and March 2022, to assemble a retrospective observational cohort of adults (over 18 years) in contact with mental health services from the most deprived communities.

### Ethics statement

Ethical approval was granted by Health Research Authority Research Ethics Committee (REC) in October 2022 (22/NS/0080). Under Regulation 5 of the Health Service (Control of Patient Information) Regulation 2002 (‘section 251 support”), this study has been approved to process confidential patient information without consent by Confidentiality Advisory Group (22/CAG/0093). The Confidentiality Advisory Group is an independent group of lay people and professionals which provides expert advice on the use of confidential patient information without consent in the United Kingdom. Data sharing agreements will be in place. Applications for NHS routine data will be made to NHS Digital via Data Access Request Service (DARS). To protect privacy and confidentiality, approval for the linkage of health data is provided under strict conditions for the storage, retention and use of the data. Only anonymised data will be shared with the approved research team for analysis in this study. CAG and REC approval letters are provided (see online supplementary appendix).

### Setting

The North East and North Cumbria (NENC) is the setting for this research, which is consistently ranked as having the highest poverty levels and the lowest health outcomes of any region in England. Participants in this research will be within the 20 GP practices identified as “Deep End” that fell into the 10% most deprived GP practices in England against the Index of Multiple Deprivation (IMD, 2019), prioritising geographical representation from Deep End GP practices across the NENC region. These GP practices have between 57.6% and 95.9% of registered patients living in the most deprived 15% of IMD Lower Layer Super Output Areas (LSOAs). The findings of this research could understand the effects of COVID-19 on deprived population and guide the development of the region’s Deep End Network.

### Base cohort

**Fig 1:**
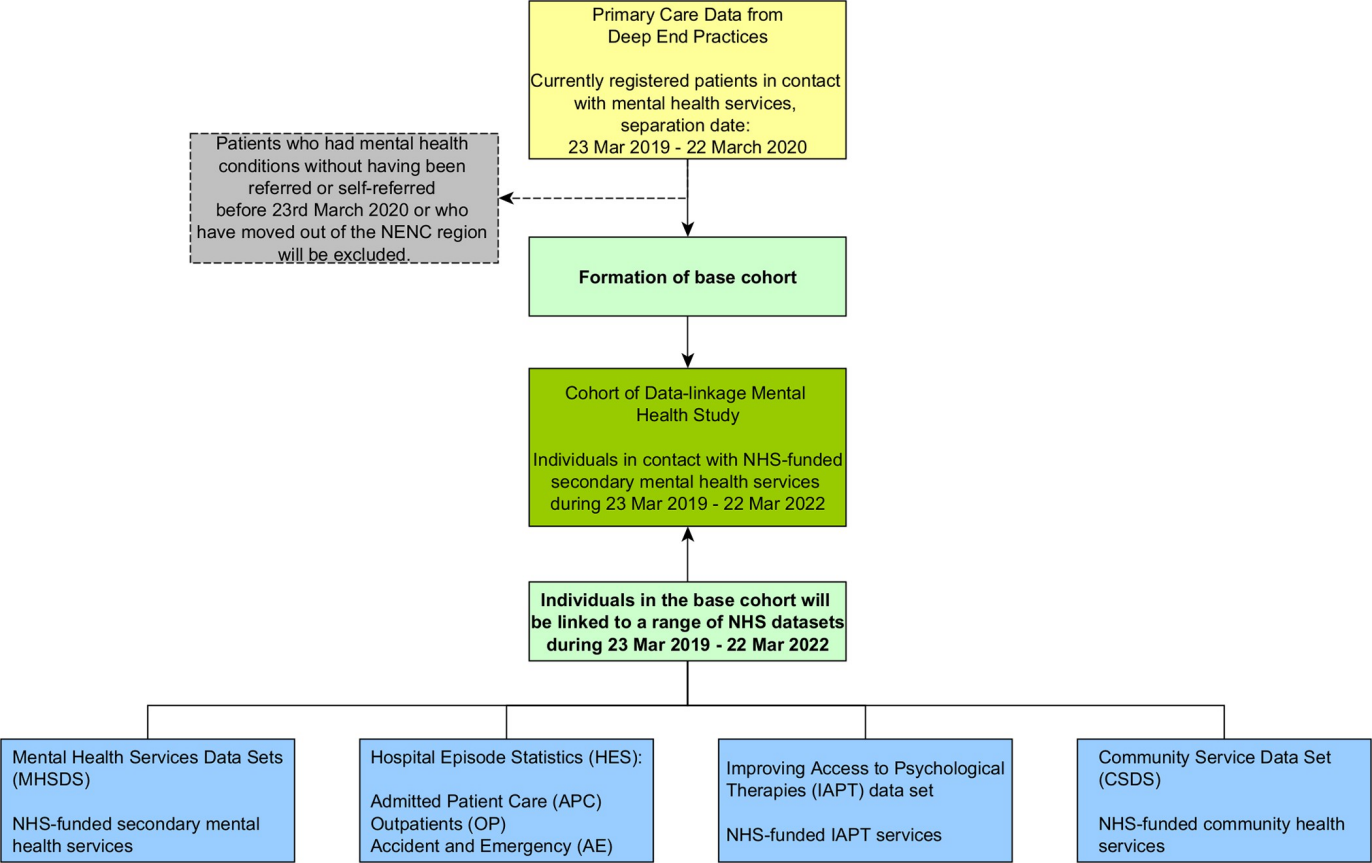
Formation of the linked datasets.

The study will include adults over 18 years because there are significant differences between the organisation of child and adolescent mental health services and adult mental health services in England. The base cohort from the Deep End GPs will consist of patients who are currently registered with the practices and who were referred or self-referred to NHS-funded secondary mental health services or Improving Access to Psychological Therapies (IAPT) services between 23 March 2019 and 22 March 2020. Data for mental health referrals is defined as instance where a patient’s care was directed to mental health services, including referrals to secondary care and community care. A flow chart of how the base cohort will be formed and the administrative data sets to be linked is presented in Fig 1. Inclusion in the cohort may be modified depending on the specific research question being addressed. Patients who had mental health conditions without having been referred or self-referred before 23^rd^ March 2020 or who have moved out of the NENC region will be excluded in this study. For those who have moved into the NENC region during the pandemic, we will examine the impact of population movement on the outcomes of interest and control for this factor in the statistical analysis in order to isolate its effect on the results. The data linkage is expected to be completed by February 2023 but is subject to timely approvals and linkage from external agencies.

### Sampling frame

We will use a total enumeration approach to include all participants fitting the inclusion criteria in each of the identified GP practices. Of the 34 local Deep End GP practices, four were in significantly rural areas, two were in urban with significantly rural areas, and 28 were in predominantly urban areas. To maximise the rural sample size, all of the six GP practices in either significantly rural or urban with significant rural areas were selected. 14 GP practices with the highest IMD scores from the 28 GP practices in predominantly urban areas were selected.

The Mental Health Statistics (England, 2021) indicated an estimated 5% of the national population had contact with NHS-funded secondary mental health services during 2020/21. From the Fingertips data (2019/20) [15], we expect 107,808 adults registered across the 20 GP practices. Therefore, we estimated a total sample of 5,390 adults in contact with NHS-funded secondary mental health services in the 20 GP practices. As a general assessment of power to detect changes in waiting time comparing before and after the COVID disruption to services, we can report power for the simplistic situation assuming a paired t-test was to be used to detect changes in waiting time. 5,390 participants would give us 95% power at the two-sided 5% significance level to detect a true difference of 0.05 standard deviations in waiting time. This sample size calculation is a guide, as in reality the data will consist of repeated measurements of the participants and more appropriate methods will be applied for the analysis which take account of the repeated measurements and nested nature of the data.

### Time periods of the COVID-19 lockdown in England

Time periods [16] studied are

- Pre-lockdown (23^rd^ March 2019 – 22^nd^ March 2020)
- First national lockdown (23^rd^ March– 3^rd^ July 2020)
- Minimal lockdown restrictions (4^th^ July– 13^th^ September 2020)
- Reimposing restrictions (14^th^ September– 4^th^ November 2020)
- Second and third lockdowns Lockdowns 2 and 3 were combined because of a short interval between them (5^th^ November– 7^th^ March 2021)
- Leaving lockdown (8^th^ March 2021–30 June 2021)
- Out of lockdown (1^st^ July 2021 – 22^nd^ March 2022)

### Routine data sources and linkage

On identifying the base cohort, North of England Commissioning Support Unit (NECS) will 1) extract primary care data from the identified GP practices; 2) send a list of identified patients with their personal information (i.e., NHS number, name, and date of birth) to request secondary and community care data from NHS Digital; 3) perform data linkage using the personal information for each patient which is a required field in all datasets; 4) create unique identifiers for all participants and will apply a pseudonymised code to the datasets; and 5) supply a pseudonymised linked data to the researchers for analysis. The linked data will be individual level data available from 23^rd^ March 2019 to 22^nd^ March 2022. NECS will retain the key for the pseudonymisation, which will not be released to the research team.

#### Primary care data

The local primary care electronic patient records (EPRs) data will be used to identify participants in contact with NHS-funded mental health services in the year prior to the lockdown. EPRs data provides varying information about patients, such as sex, ethnicity, diagnoses, symptoms, observations, test results, medications, allergies, immunisations, referrals, recalls, appointments, and information about physical, mental and sexual health, including staff who have treated patients. Participants’ NHS numbers, name, and date of birth are required for the identified cohort to link the primary care data with personal confidential data from other care settings in order to analyse patient care across pathways.

#### Secondary care–Mental Health Services Data Set (MHSDS)

This national dataset collates monthly individual-level data on all patients in England who had contact with any type of secondary mental health services provided and/or funded by NHS England. This includes voluntary and involuntary inpatient treatment, outpatient attendance, community mental healthcare, and other episodes of secondary mental healthcare.

MHSDS data are based on spells of care for individual patients. We will use the dates on which specific care spells start and end to determine duration of service use and hospital (re)admissions. MHSDS provides data on a range of patient characteristics (e.g., age, sex, ethnicity, etc.) and data on clinical characteristics (e.g., diagnosis, care clusters [17], and Health of the Nation Outcome Scales [18]). Additional patient characteristics such as marital, employment and accommodation status may be present in the dataset, although previous research noted high levels of missing data [19].

#### Secondary care–Improving Access to Psychological Therapies (IAPT) data set

The NHS-funded IAPT services that is not covered by the MHSDS were launched in 2008 to improve the quality and accessibility of mental health services in England [20]. Its focus is on therapies like cognitive behavioural therapy, counselling and self-help support, collectively known as ‘talking therapies’, for working-age people experiencing common mental health problems such as anxiety and depression. People can be referred to IAPT by their GP, or they can self-refer. IAPT aims to expand access to psychological therapies to 350,000 more adults each year by 2020/21 and reach 25% of those with common mental health problems every year [21].

IAPT data include waiting time between referral and entering treatment, the number of sessions, length of treatment, the number of cancellations and non-attendance, the recording of problem descriptor information (an ICD-10 code), the index of multiple deprivation of the catchment area of the service, and patient outcomes.

Some patients received just an assessment and advice or signposting. Therefore, a course of IAPT treatment in this study is defined as two or more treatment sessions. However, this ignores patients receiving only one treatment session and those with less severe presentations to services, and there may be important changes in the number of patients receiving only one session that might further influence changes in patient outcome, as suggested by Clark, Canvin [22].

Two IAPT-defined patient outcomes, recovery and reliable improvement, will be considered in this study, both of which are used in national IAPT reporting [23]. The former is defined in IAPT as moving from scoring above caseness for either depression or anxiety at the start of treatment to scoring below caseness on measures of both depression and anxiety symptoms at the end of treatment. The latter is defined as a reduction in symptom scores above the error of measurement for the depression and anxiety measures used.

#### Secondary care–Hospital Episode Statistics (HES)

The Hospital Episode Statistics (HES) dataset provides a centralised repository of secondary care admission and appointment records within NHS hospitals and independent sector health care providers where the treatment was commissioned by the NHS in England since 1990. It is comprised of four datasets: Admitted Patient Care (APC), Outpatients (OP), Accident and Emergency (A&E), and Critical Care (CC). The data cover diverse topics including diagnosis, maternity, mortality, mental health, types of therapies, length of treatment, Indices of Multiple Deprivation (IMD), service providers, organisations, and regional geographical location.

*Hospital admission records (APC)*. Hospital admissions include episodes of treatment that require the use of a hospital bed. The full documentation can be found in the HES APC data dictionary.

*Outpatient records (OP)*. The HES OP dataset records outpatient appointments in English NHS hospitals and English NHS commissioned activity in the independent sector.

Data completeness is an issue in some OP fields. While ‘attendance type’, ‘source of referral’ and ‘main specialty’ have high rates of completeness (>98%), the outcome variable is less complete (95%) and fields such as primary diagnosis (5%) and main procedure (26%) have low levels of completeness.

*Accident and Emergency records (A&E)*. The HES A&E dataset records attendance at Accident & Emergency departments. Within the NHS, A&E departments provide services for those seeking urgent care for injury and illness. Major A&E departments receive new patients on a continual basis and care is consultant led. The HES A&E dataset also includes attendance records for specialty A&E departments, walk-in centres and minor injury units.

#### Community Service Data Set (CSDS)

This national dataset holds patient-level data on all individuals in contact with NHS-funded community health services in England. This data includes all services referred to for community care, including NHS Trusts, health centres, schools, mental health trusts, and local authorities. The key fields include personal and demographic information, social and personal circumstances, diagnoses including long-term conditions and disabilities, care events plus screening activities, and scored assessments.

### Aggregate data

GP practice-level data will be extracted from Fingertips [15], which is a publicly accessible web tool (https://fingertips.phe.org.uk/) maintained by Public Health England. It provides access to a wide range of local public health data presented as thematic profile. Practice-level factors will include the GP practice profiles, mental health profiles, and overall achievement.

### Measurements of outcomes

Changes to mental health services utilisation in response to COVID-19 and their impact on health outcomes will be identified from a rapid review of the literature [24]. In addition, the study may investigate selected outcomes for which there is unknown evidence of an association with service utilisation.

Mental health service measures will include:

- Referrals to new services (e.g., type of referrals, waiting times, progression from low to high intensity treatment)
- Waiting time is defined as number of days that a patient waited in a referral to treatment period.
- Access to mental health services
- We define “access to mental health services” as having at least one recorded contact (whether attended or not) for a mental health reason.
- Modes of contact with services
- Pattern of mental health services utilisation

Defined as duration of service use (e.g., the number of GP consultations, the number of admitted (inpatient) days, the number of days with contact with a healthcare professional)

- Treatment adherence/completion (i.e., the number of attended, cancelled and non-attended appointments)

Non-attendance is considered as an inefficient use of health service resources

- Prescription of antidepressants

Health outcome measures related to mental health services will include:

- Mental health (e.g., depression, PHQ-9; anxiety, GAD-7; HoNOS)

Depression symptom severity was measured using the Patient Health Questionnaire 9-item version (PHQ-9) [25], where scores of 10 or above indicate caseness for depression, and a reduction of 6 or more points on the scale indicates reliable improvement in depression symptoms.

The Generalized Anxiety Disorder Scale 7-item (GAD-7) [26] is the main measure of anxiety symptoms used in IAPT services. Caseness is defined as scores of 8 or above, and a reduction of 4 or more points indicates reliable improvement.

The Health of the Nation Outcome Scales (HoNOS) is a clinician rated instrument comprising 12 simple scales measuring behaviour, impairment, symptoms and social functioning for those in the 18–64 years old age group.

- Physical health (e.g., heart disease, diabetes, or diabetes)
- Self-harming
- Psychotropic medication (outcome or service/treatment use?)
- Common mental disorder (CMD) symptoms
- Mortality: premature mortality, time to death
- Time to first readmission
- Use of A&E, A&E readmissions within 30 days
- Morbidity measured as unplanned assessment by emergency care and inpatient admission

### Measurements of confounders/mediators

Individual-level clinical characteristics will include:

- Diagnoses
- Prescription
- Hypertension
- High blood cholesterol
- Long-term conditions/co-morbidities

Individual-level characteristics will include:

- Socio-demographics (e.g., age, gender, ethnicity, etc.)
- Smoking status
- Alcohol intake
- BMI
- Physical activity

GP practice-level characteristics will include:

- List size
- percent of satisfaction
- percent of a long-standing health condition
- percent of caring responsibility
- percent of being in paid work or in full-time education
- percent of being unemployed

### Planned statistical analyses

In all analyses, multiple confounding variables will be controlled for as appropriate. The below analyses will be undertaken for the total cohort and subgroups (e.g., age, gender, ethnicity, or socioeconomic backgrounds). All analyses will be carried out using Stata version 17.0 [27].

Descriptive analysis will be used to examine socio-demographic characteristics (e.g., age, sex, etc) and clinical characteristics (e.g., diagnosis, prescriptions, etc) for the cohort within the 12-month period prior to the first lockdown. Comparisons will be made using the t-test, rank-sum and chi-square where appropriate. A p<0.05 is considered as significant. If significant health service utilisation imbalance is detected on a particular variable at patient level, and that variable is correlated with outcomes at a level of 0.3 or higher, the variable will be included as a covariate in regression analyses.

Multi-level regression analysis will be used to quantify mental health service utilisation among the cohort at the different time periods of the lockdown and to assess individual- and practice-level characteristics as predictors of health service utilisation because of the hierarchical nature of the data. A three-level model will be constructed (patients clustered within practice, clustered within time period). The model makes two assumptions: 1) that lockdown rules are similar across different geographic areas, and 2) that there are random effects across practices, which allows each practice to have its own dynamics while still taking all information into account when estimating model parameters.

In this study, latent growth models will be applied within a structural equation modelling framework to analyse the relationship between changes in health service utilization and health outcomes. The growth trajectories will be divided into segments representing each time period, and separate growth curves will be fitted to each segment simultaneously. Additionally, the change process of two or more variables will be examined to determine how they are related to one another (e.g., the change in waiting time and the change in health outcomes). Individual and practice-level characteristics will also be included as predictors of health outcomes.

### Comparative analyses

This study focuses on people living in the most deprived areas, and we intend to compare their situation to that of people living in the least deprived areas if we can access data on people living in the least deprived areas. This part of the project is at earlier stage of development and the detailed research is still subject to funding and approval. Comparative analysis offers the prospect of distinguishing the contributions of individual factors to geographic variation in health outcomes.

### Missing data

For primary analyses, missing data will not be imputed if the values are missing completely at random (MCAR). If it is concluded that data are not MCAR, multiple imputation using chained equations will be performed by creating 10 impute data sets under the assumption of that the missing data are missing at random (MAR). We will use the stratification-variable as well as other known predictive outcomes in the multiple imputation to estimate the missing values. The analysis will be presented as a pooled summary of the results from the 10 datasets. It is not uncommon that missing data and missing variables are discovered only after initiation of research using electronic health record data, making it possible for us to deviate from our original research plan. If so, details of deviation from the protocol will be reported and reasons for the deviation and the implications on the research and conclusions will be discussed. Sensitivity analyses may be reported to evaluate the potential impact of missingness of data and representativeness of the study population.

## Patient and public involvement (PPI)

In this study, PPI partners are involved in the planning, implementation, evaluation, and dissemination process. The participation of patients, professionals, and support organisations has been crucial in the design of the study. A advisory group will provide guidance to the project to maximise its relevance and impact. The group will consist of mental health service users, their carers, health professionals (inc. managers and clinicians), policy makers and commissioners. Service users will be recruited by the Clinical Research Network. Recruitment will ensure diversity of age, gender, and ethnicity. The advisory group will help us interpret the findings of our study and ensure that analyses and dissemination are relevant to the needs of stakeholders.

## Dissemination

Findings will be reported in accordance with the **RE**porting of studies **C**onducted using **O**bservational **R**outinely-collected health **D**ata statement (RECORD) [28] and **G**uidelines for **A**ccurate and **T**ransparent **H**ealth **E**stimates **R**eporting (GATHER) [29], where appropriate.

Research findings will be disseminated via websites, at scientific conferences and in peer-reviewed journals. Emerging findings and learning captured will also be shared with underserved communities, GPs, and commissioners across the region to guide the development of the region’s Deep End Network, improve services and reduce health inequalities.

## Discussion

### Main findings

This study aims to examine the impact of the COVID-19 pandemic on patterns of NHS services utilization in the most deprived communities in England. It will determine whether these patterns are associated with health-related outcomes and wellbeing. The findings of this research could provide valuable insights into how the pandemic has affected the utilisation of NHS services in disadvantaged communities and could inform policy and practice in the field of public health. The study could also help to improve our understanding of individual and situational factors that predict mental health-related outcomes and wellbeing, potentially leading to more personalized interventions in practice. Additionally, the patterns of healthcare utilisation identified in this study could improve our knowledge of patient needs and enhance healthcare delivery for disadvantaged communities. This research could serve as a starting point for further studies on this topic, potentially leading to more effective interventions and policies in the future.

### Strengths and limitations of the study

With data spanning multiple years and the ability to link records across different services, this study makes it possible to explore the healthcare utilisation history of patients across multiple healthcare sectors both before and after the lockdown. This study can identify the types of referrals to healthcare services made at different points in time facilitating assessment of health service usage and recommendations for improving patient care pathways, and thus maximise clinical efficiency and efficacy in the use of resources. The study findings will be assessed and presented to direct policy relevance, and we plan to share them directly with those bodies working in these areas at the earliest opportunity.

However, data obtained from routinely collected data systems often require careful interpretation with respect to their quality, validity, timelines, bias, residual confounding and statistical stability [30]. For example, missing data are important sources of bias [31]. Routinely collected administrative data contain limited contextual information and represent an underestimate of total health outcomes for these individuals. Further, intervention and treatment for mental health conditions are not wholly captured across these data sources and may impact health outcomes.
